# Label-free detection of single cell by ZnO/Graphene/AgNPs hybrid microcavity enhanced Raman scattering

**DOI:** 10.3389/fchem.2025.1636525

**Published:** 2025-06-25

**Authors:** Yaqi Shan, Jihao Wu, Kai Cui, Juan Yang

**Affiliations:** School of Chemical Engineering and Technology, Xi’an Jiaotong University, Xi’an, Shaanxi, China

**Keywords:** label-free detection, quantitative detection, single cell, ZnO/Graphene/AgNPs, hybrid microcavity

## Abstract

Ultrasensitive detection of *Escherichia coli* (*E. coli*) is important for early diagnosis of foodborne diseases. Current analytical techniques face limitations in performing label-free quantification of viable bacterial cells at single-cell resolution. Herein, a hybrid enhanced Raman scattering probe was constructed by assembling a layer of graphene and silver nanoparticles on a hexagonal ZnO microrod (ZnO/Graphene/AgNPs) for ultrasensitive analysis of pathogens directly. Ultimately, quantitative detection of *E. coli* was successfully carried out with excellent detectability from 1 cell mL^−1^ to 1 × 10^8^ cells mL^−1^. It provided a detection limit as low as 4.57 × 10^−2^ cell mL^−1^ for *E. coli*. This can be attributed to the synergistic effect of different components, i.e., the ZnO microrod provides natural whispering gallery mode (WGM) microcavity which enhances light-matter interaction through multiple total internal reflections, graphene assists charge transfer, and AgNPs produce surface plasmons. These three enhancement factors are integrated to achieve label-free ultrasensitive detection. This work highlights a label-free approach for a variety of clinically relevant biomolecules to achieve early diagnosis of the disease.

## 1 Introduction

The timely and precise identification of pathogenic microorganisms holds critical importance for enhancing clinical outcomes, controlling disease transmission, and tracing infection origins (Cui et al., 2024). Notably, during initial disease phases, merely a minute fraction of cells possess specific diagnostic markers signaling biological abnormalities. However, regular pathogen detection usually requires thousands of cells in the analytes, potentially obscuring crucial pathological indicators through population averaging. Quantitatively examining each cellular component in a large number of cells would be beneficial to effective differentiation between diseased and healthy cells, thereby strengthening diagnostic accuracy (Xi et al., 2024). Therefore, ones hope to catch clear behaviors in complex systems (Li and Xie, 2011) from a few cells even single cell level (Zhou et al., 2024), necessitating advancements in detection sensitivity and the reduction of the detection limit. In recent years, label-free ultrasensitive biosensors have been paid attention in the field of life science research and diagnostics because of their advantage for *in situ* and real-time detection in point-of-care testing applications (Needham et al., 2024).

Whispering gallery mode (WGM) microcavity exhibits exceptional sensitivity to minute refractive index variations in surrounding media through enhanced light-matter interactions enabled by continuous total internal reflections at resonator interfaces (Yu et al., 2021). So far, the high sensitivity and low detection limit have been realized in microdisk (Armani et al., 2007), microsphere (Guan et al., 2024), microring (Toropov et al., 2021), microbottle (Zhu et al., 2020) WGM cavities, which have even been able to detect single particle or molecule, such as a virus or nucleic acid (Tsalsabila et al., 2024). However, there are challenges in living cell detection, especially for single cells or bacteria. Contemporary SERS approaches for living cells or bacteria detection primarily employ two methodologies: targeted labeling strategies and direct spectral acquisition techniques. Conventional label-based SERS protocols involve functionalizing metallic nanoparticles with both Raman-active reporter molecules and biological recognition elements to generate specific spectral signatures (Wang et al., 2013). Nevertheless, the exposed surfaces of metallic nanoparticles tend to adsorb extraneous biomolecules from complex biological matrices, potentially compromising signal stability (Liu et al., 2010). Additionally, the conjugation process requiring specific antibodies, aptamers, or molecular ligands to functionalize SERS tags introduces operational complexity (Szlag et al., 2018). Label-independent SERS methodologies have emerged as promising alternatives for bacterial analysis, offering inherent sensitivity combined with information-rich spectral fingerprints that advance cellular characterization techniques (Zhang et al., 2023). The remarkable label-free strategy involves either colloidal mixing of noble metal nanostructures with bacterial suspensions or substrate-mediated nanoparticle assembly for microbial adhesion (Kant et al., 2024). Many studies have achieved successful pathogen identification through these approaches, but quantitative analysis remains suboptimal (Wang et al., 2015). However, it was still very difficult to achieve single-cell information in living cells or bacteria, though many researchers have tried to improve the analytical detection limit by various methods (Li et al., 2025; Zhang et al., 2024). Facing the challenge, one has to solve two issues: how to enhance the signal-to-noise ratio, and how to guarantee the precision.

The strategy in this paper is to enhance the Raman signal by integrating the optical resonator, localized surface plasmon resonance (LSPR) phenomena, and charge transfer effects in a WGM hybrid microcavity, which has been strongly supported by previous reports (Zhu et al., 2019). Briefly, silver nanoparticles and graphene were assembled on an individual ZnO microrod to construct a hybrid SERS substrate for the living bacteria test. In this design, the Ag NPs would further intensify light-matter interactions through plasmon coupling with ZnO microcavity and graphene also contributes to the Raman signal through its favorable physical adsorption and chemical enhancement based on charge transfer. As a result, single cell of *E. coli* (*E. coli*) could be detected directly in the culture medium without any label and further achieved excellent quantitative detection ability.

## 2 Experimental section

The fabrication of ZnO microrods on the Si surface was achieved through a vapor-phase transport method following established protocols. Firstly, a single ZnO microrod was selected and carefully positioned on the Si surface. Secondly, a graphene monolayer was precisely transferred onto the ZnO microrod to form a hybrid ZnO/Graphene microcavity structure. Finally, a controlled sputtering process lasting 15 s deposited AgNPs simultaneously onto multiple surfaces including the hybrid microcavity, bare ZnO, and Si substrates. Furthermore, the fabrication process of ZnO microrods and ZnO/Graphene/AgNPs microcavity, as well as the optimization of sputtering time, were presented in (Supplementary Figures S1, S2). Six distinct areas comprising different material combinations 1) ZnO/Graphene/AgNPs, 2) ZnO/AgNPs, 3) ZnO, 4) Si/Graphene/AgNPs, 5) Si/AgNPs and 6) Si were systematically created on a single substrate for comparative analysis of SERS enhancement mechanisms involving ZnO, graphene, and silver nanoparticles.

The structural features of ZnO, AgNPs, and ZnO/Graphene/AgNPs hybrid structures were examined through field emission scanning electron microscopy (SEM) coupled with an X-ray energy dispersive spectrometer (EDS). Optical properties were conducted by a 325 nm fs (fs) laser. For SERS analysis, measurements employed a Raman spectrometer featuring a 532 nm wavelength laser operating at 2 mW power with 10 s integration periods. Raman spectral data collection covered the characteristic molecular vibration range of 600–1800 cm^−1^. *E. coli* was cultured in lysogeny broth (LB) for 24 h and its concentration was calculated by colony counting method. Prior to spectral acquisition, a certain amount of *E. coli* solution is dropped onto the surface of the SERS substrate for subsequent Raman signal detection.

## 3 Results and discussion

**FIGURE 1:**
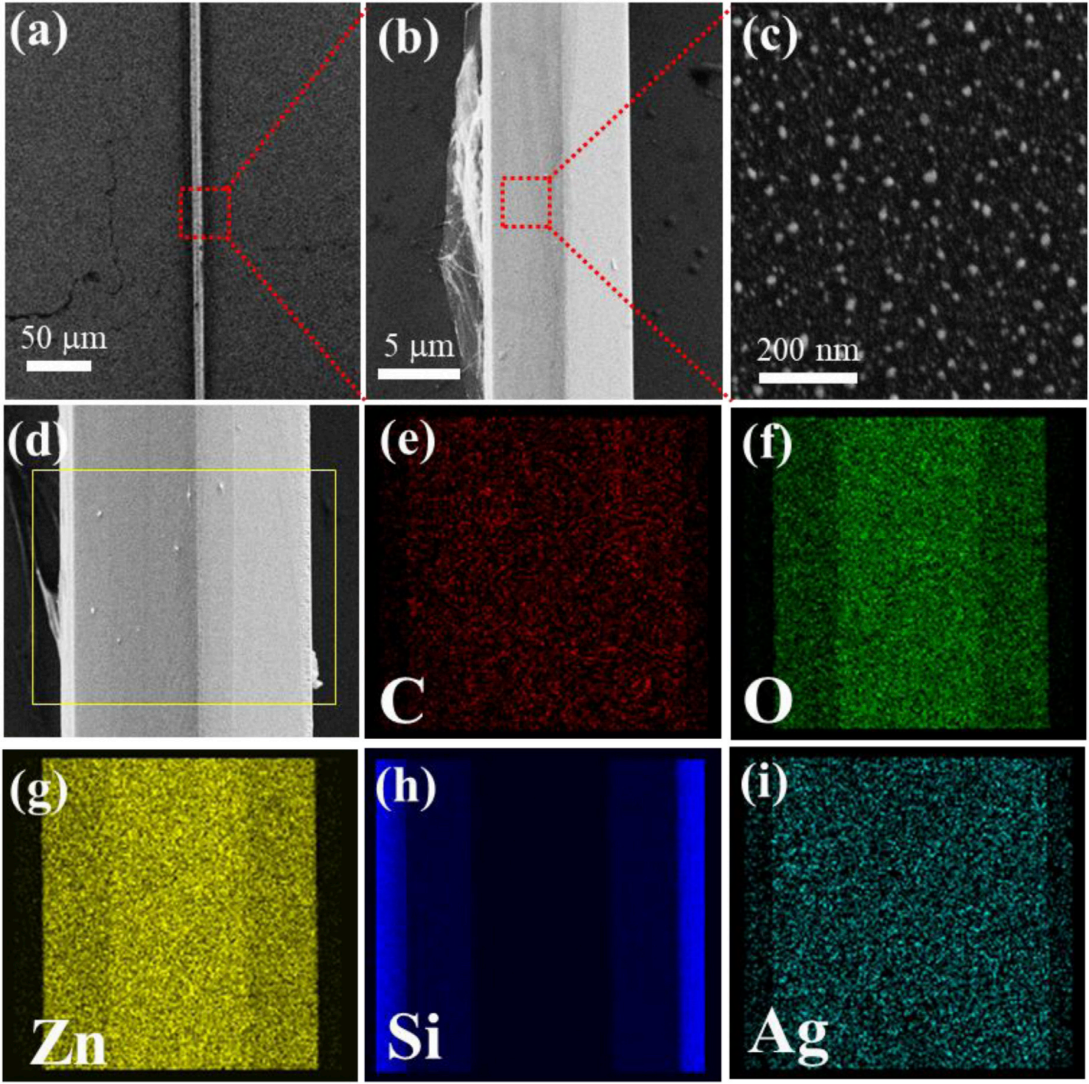
**(a–c)** Display SEM micrographs of ZnO/Graphene/AgNPs. **(d-i)** The corresponding element mappings obtained from the yellow outlined rectangular region.

A ZnO microrod with a smooth hexagonal structure was first put onto the Si surface (Supplementary Figure S1), with a diameter of 9.805 μm. Figure 1a shows that the ZnO microrod surface demonstrates cover with graphene successfully. The existence of graphene was confirmed by using Raman spectroscopy (Supplementary Figure S3). It can be seen that the ZnO microrod exhibits a typical Raman peak associated with the wurtzite structure at 438 cm^−1^, while the peak at 303 cm^−1^ is related to defects in ZnO. Additionally, two characteristic Raman peaks of graphene, namely, the G peak and 2D peak, appear at 1568 cm^−1^ and 2677 cm^−1^, respectively. It is noteworthy that the intensity ratio of I_2D_/I_G_ is greater than 1, indicating that the graphene possesses a monolayer structure. Figure 1b shows that the ZnO microrod surface demonstrates cover with graphene and AgNPs successfully. Figure 1c details the uniform distribution of AgNPs across the ZnO/Graphene/AgNPs, with particle diameters averaging 20 nm. The coexistence of C, Ag, Zn, O, and Si elements within the ZnO/Graphene/AgNPs hybrid substrate was verified through elemental mapping analysis (Figures 1d-i; Supplementary Figure S4). Distinct spatial distributions emerge, with Zn and O signals corresponding precisely to the microrod’s architecture while Si originates from the underlying substrate. Additionally, C and Ag elements exhibit uniform surface dispersion patterns across the hybrid structure, confirming the successful integration of all components.

**FIGURE 2:**
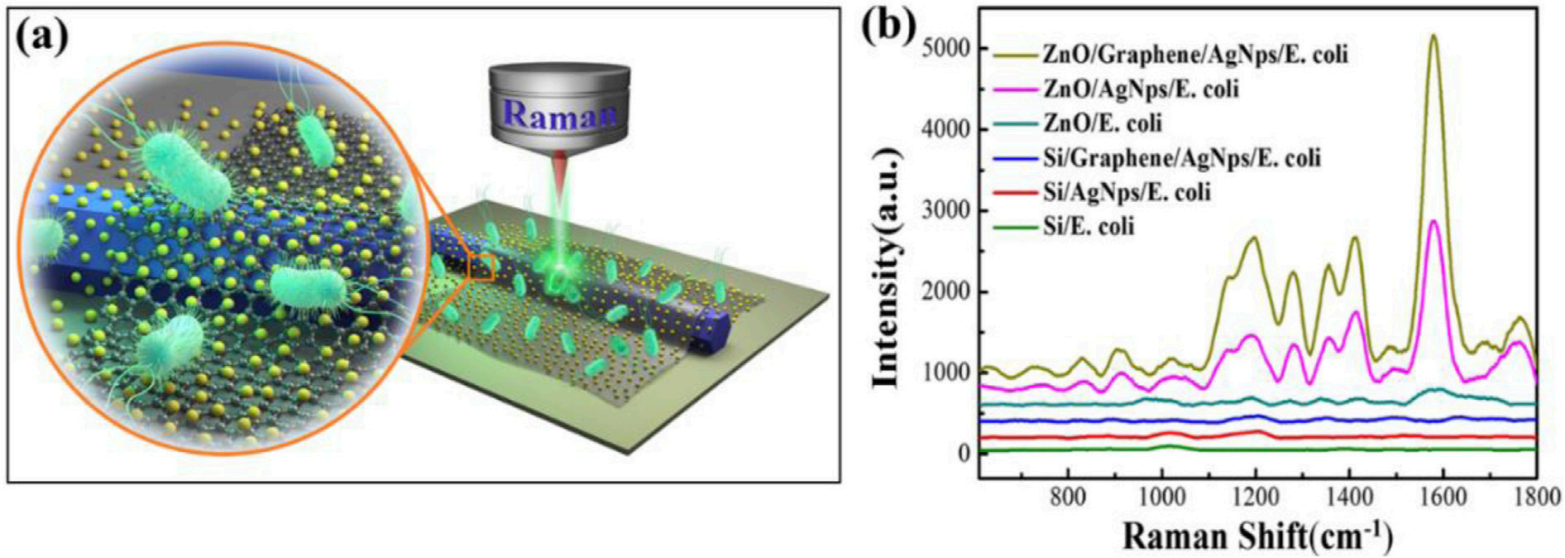
**(a)** Schematic diagram of Raman detection of *E*. *coli*. **(b)** Comparative Raman spectral profiles of *E. coli* obtained from various substrates: ZnO/Graphene/AgNPs, ZnO/AgNPs, ZnO, Si/Graphene/AgNPs and Si/AgNPs hybrid microcavity regions, along with the reference Si substrate.

To explore the enhanced performance of ZnO, graphene, and AgNPs in SERS hybrid substrate, six distinct detection zones (designated 1–6) were established with varying material combinations: ZnO/Graphene/AgNPs, ZnO/AgNPs, ZnO, Si/Graphene/AgNPs, Si/AgNPs and Si substrate (Figure 2a). *E. coli* is the detection target used for demonstrating the enhancement effect of SERS. Comparative Raman spectral signals of *E. coli* from different regions as shown in Figure 2b; It can be observed that AgNPs and Graphene can enhance the Raman signal of *E. coli* in silicon-based configurations (Si, Si/AgNPs, Si/Graphene/AgNPs), but the enhancement effect is not obvious (Supplementary Figure S5). Notably, ZnO demonstrated Raman signal amplification compared to Si. Crucially, AgNPs decoration induced 10-fold Raman signal intensity enhancement over ZnO, and subsequent graphene integration doubled this amplification. In total, the ZnO/Graphene/AgNPs hybrid microcavity achieved more than 20-fold Raman intensity enhancement. This synergistic enhancement mechanism arises from three primary factors: light-matter interactions through ZnO WGM, charge transfer mediated from graphene, and localized surface plasmon resonance from AgNPs (Lu et al., 2015; Lu et al., 2014). Furthermore, the lasing performance and the finite-difference time-domain (FDTD) simulation of hybrid microcavity were displayed in (Supplementary Figures S6, S7). It was found that the smooth-surfaced ZnO microrods serve as WGM microcavity, while monolayer graphene and AgNPs act as surface plasmons, enabling greater light localization at the surface of the hybrid microcavity and significantly enhancing its laser performance.

**FIGURE 3:**
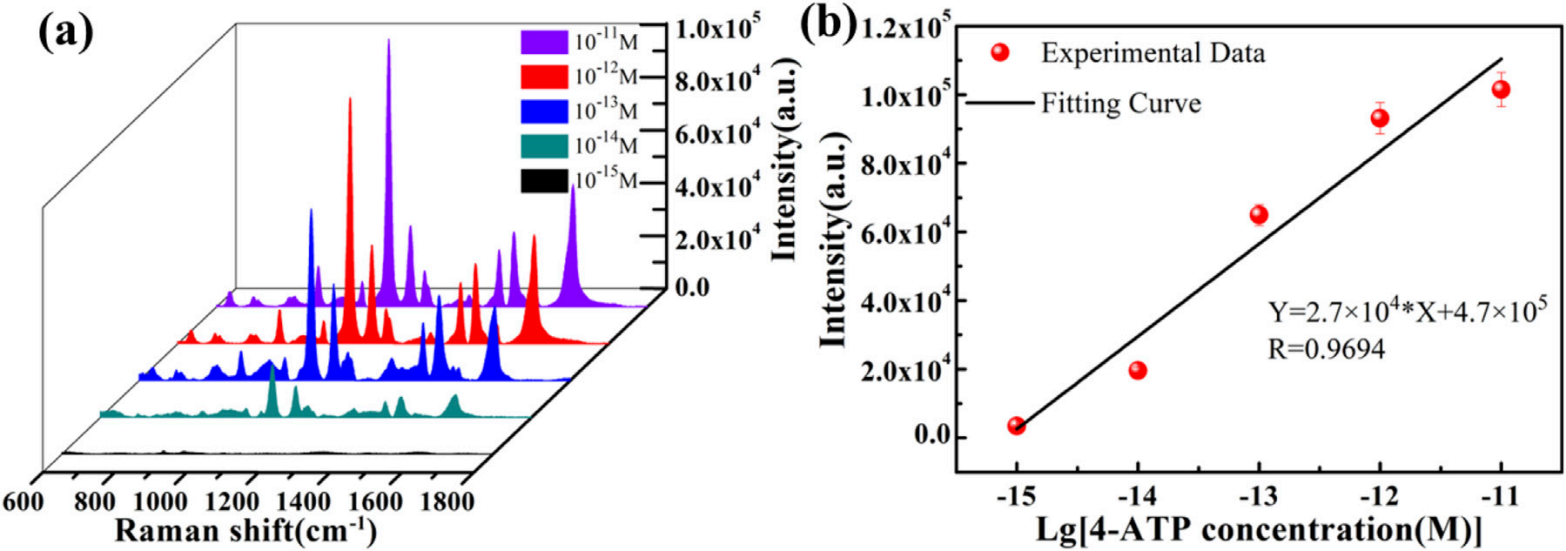
**(a)** Raman spectra of 4-ATP with different concentrations identified by the ZnO/Graphene/AgNPs hybrid microcavity. **(b)** The dependence of Raman peak intensity at 1080 cm^−1^ on 4-ATP concentrations.

To verify the enhancement effect of the ZnO/Graphene/AgNPs hybrid microcavity on Raman signals, 4-aminothiophenol (4-ATP) was employed as the Raman probe molecule. Raman tests were conducted on 4-ATP with varying concentrations using the SERS substrate of the ZnO/graphene/AgNPs hybrid microcavity, as illustrated in Figure 3a. This can be used to evaluate the dependence of the Raman signal peak intensity. Results indicated that as the concentration of 4-ATP increased from 10^–15^ M to 10^–11^ M, the Raman signal intensity of the ZnO/graphene/Ag NPs hybrid microcavity gradually increased. Surprisingly, even at a low concentration 10^–15^ M, the characteristic peaks of the Raman spectrum of the SERS hybrid substrate were still clearly visible. As the concentration of 4-ATP increased, more substances interacted with light, causing the number of photons localized on the ZnO surface to gradually increase, which in turn led to a gradual increase in the Raman signal intensity. A series of the strongest Raman peaks were selected as the y-axis, and the logarithm of the corresponding 4-ATP concentration was taken as the x-axis. A linear fit was performed, and the fitting result is shown in Figure 3b. It was found that there was a good linear relationship between them, and the linear regression equation was Y = 2.7 × 10^4^*X + 4.7 × 10^5^, with a correlation coefficient of 0.9694 and a detection limit (LOD) of 3.6 × 10^−16^ M. Additionally, the enhancement factor (EF) of the ZnO/Graphene/AgNPs hybrid microcavity SERS substrate was evaluated within the above detection range. Using a Si substrate as the blank control substrate, the EF of the SERS substrate was estimated based on Equation 1:

$$\text{EF}=\left(I_{\text{SERS}}\times N_{\text{Bulk}}\right)/\left(I_{\text{Bulk}}\times N_{\text{SERS}}\right)$$ (1)

Here, I_SERS_ represents the Raman signal intensity at 1080 cm^−1^ of 10^–15^ M 4-ATP on the ZnO/Graphene/AgNPs hybrid microcavity, and I_Bulk_ represents the Raman signal intensity at 1080 cm^−1^ of 10^–3^ M 4-ATP on the Si substrate. Additionally, N_SERS_ and N_Bulk_ are the number of 4-ATP probe molecules at the corresponding concentrations. Through calculation, the EF of the ZnO/Graphene/AgNPs hybrid microcavity is 4.92 × 10^12^, indicating that this SERS substrate has extremely high sensitivity. Based on this, it can be concluded that the ZnO/Graphene/AgNPs hybrid microcavity can be used as an effective and ultrasensitive tool for quantitative detection of Raman signals.

**FIGURE 4:**
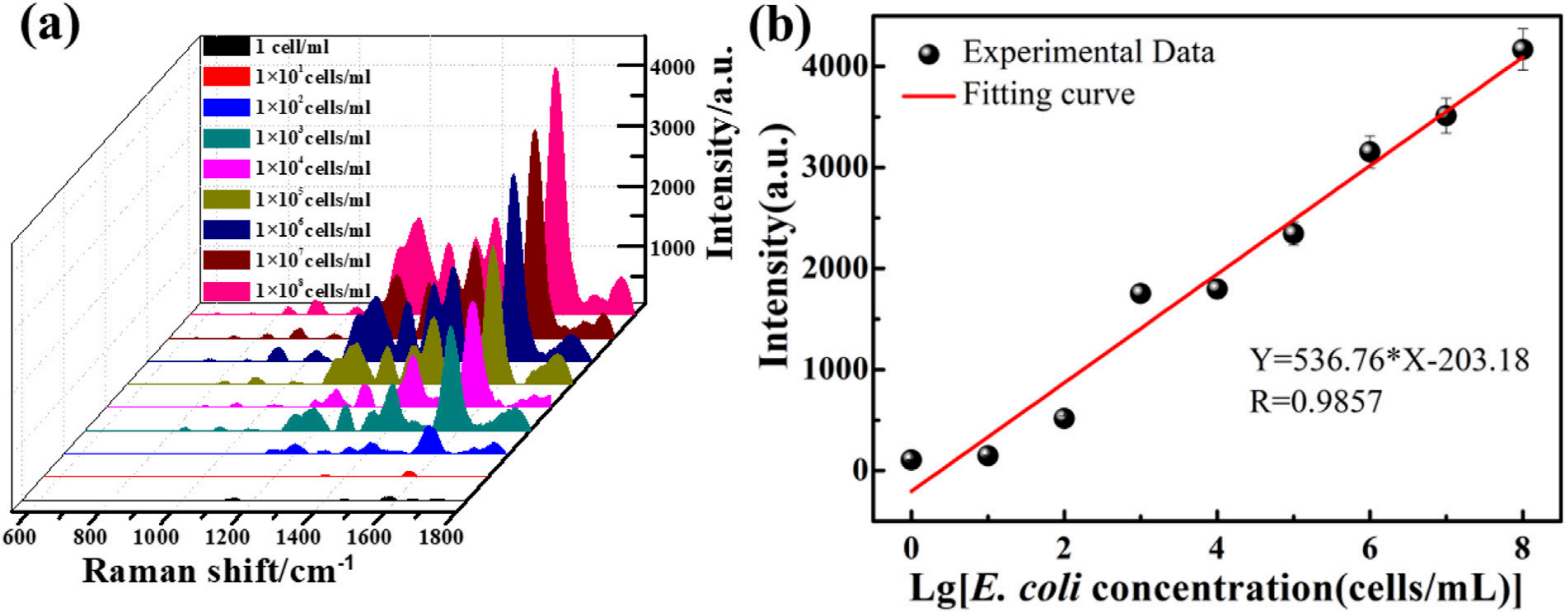
**(a)** Raman spectra of *E. coli* with different concentrations identified by the ZnO/Graphene/AgNPs hybrid microcavity. **(b)** The dependence of Raman peak intensity at 1579 cm^−1^ on *E. coli* concentrations.

As discussed above, the ZnO/Graphene/AgNPs hybrid microcavity is ideal for SERS-based ultrasensitive biosensors. It has also been demonstrated in previous work that the hybrid microcavity exhibits a remarkable sensitivity, achieving femtomolar-level detection with an EF reaching 0.95 × 10^12^ while maintaining an ultralow detection threshold of 10^–15^ M for both rhodamine 6G and dopamine.^20^ As a practice function, the hybrid biosensor was employed to detect *E. coli* with different concentrations from 1 cell mL^−1^ to 1 × 10^8^ cell mL^−1^, and the results are presented in Figure 4a. The Raman spectra showed that with the increase of *E. coli* concentration, the characteristic peaks associated with it gradually increased. Quantitative analysis in Figure 4b demonstrates the 1579 cm^−1^ peak intensity’s concentration-dependent behavior. The results can be shown that the Raman signal intensity of ZnO/Graphene/AgNPs/*E. coli* were different, which allowed us to distinguish between the presence and absence of *E. coli*. The Raman signal intensity of ZnO/Graphene/AgNPs/*E. coli* is gradually increased when increasing the concentration of *E. coli*. The calibration curve for *E. coli* is Y = 536.76lgC-203.18 (R = 0.9857), establishing a LOD of 4.57 × 10^−2^ cell mL^−1^. The standard deviation (S) for 13 parallel measurements on blank samples is about 8.18. The LOD for the method is calculated using the formula LOD = 3 S/K (the slope K = 536.76). Notably, when the detection concentration was 1 cell/ml, there was only one bacterium present in the field of view. Therefore, we collected the spectrum by focusing the light spot on a single bacterium, and the result is shown in Supplementary Figure S8.

## 4 Conclusion

In summary, single-cell and quantitative detection of *E. coli* was successfully carried out based on the ZnO/Graphene/AgNPs hybrid microcavity. A layer of graphene and silver nanoparticles was assembled on a hexagonal ZnO microrod to construct a hybrid microcavity-enhanced Raman scattering probe for ultrasensitive analysis on *E. coli* directly. The Raman scattering intensity of *E. coli* on the ZnO/Graphene/AgNPs was enhanced approximately 2-fold compared to ZnO/AgNPs and 10-fold superiority over Si/Graphene/AgNPs. Ultimately, quantitative detection of *E. coli* was successfully carried out in the range of 1 cell mL^−1^ to 1 × 10^8^ cells mL^−1^, achieving a detection limit as low as 4.57 × 10^−2^ cell mL^−1^. The high sensitivity of the Raman signal for this designed hybrid substrate was ascribed to the following three reasons. First, the hexagonal ZnO microrod provided a natural WGM microcavity, which constructed a more advantageous coupling configuration between the excitation light and the analyte molecule. Second, the chemical mechanism is based on a charge transfer between the adsorbed molecules and the graphene. Third, LSPs of AgNPs will enhance the Raman signal due to its characteristics of the altitudinal space confinement and near-field enhancements. Three synergistic mechanisms contributed to this enhancement performance: 1) The intrinsic WGM microcavity structure of ZnO microrods optimized light-matter interactions through efficient photon confinement. 2) Graphene-mediated charge transfer processes enhanced molecular polarization at the analyte-substrate interface. 3) Plasmonic AgNPs generated intense electromagnetic field enhancements through LSPR effects, particularly through spatial confinement and near-field amplification phenomena.

## Data Availability

The original contributions presented in the study are included in the article/Supplementary Material, further inquiries can be directed to the corresponding authors.
