# Supplementary material for: Label-free detection of single cell by ZnO/Graphene/AgNPs hybrid microcavity enhanced Raman scattering
Source: Front Chem. 2025 Jun 25;13:1636525. doi: 10.3389/fchem.2025.1636525 (PMC12237927; doi:10.3389/fchem.2025.1636525)
Supplement: Supplementary file 1 [file DataSheet1.docx]

Supplementary Material

Label-free detection of single cell by ZnO/Graphene/AgNPs hybrid microcavity enhanced Raman scattering

Yaqi Shan^1^, Jihao Wu^1^, Kai Cui^1*^, Juan Yang^1*^

^1^ School of Chemical Engineering and Technology, Xi’an Jiaotong University, Xi’an, Shaanxi, 710049, P. R. China.

*** Corresponding authors:**Kai Cui

E-mail: cuikai212@xjtu.edu.cn

Juan Yang

E-mail: juanyang@xjtu.edu.cn

**Experimental section**

The specific process for preparing ZnO microrods on a Si substrate using chemical vapor deposition is as follows: Firstly, thoroughly mix 2.8 g of high-purity ZnO (99.99%) powder and 0.2 g of high-purity carbon powder (99.99%) in a mortar. Take 0.8 g of this mixture and place it in a quartz boat. Invert a clean Si substrate onto the mixture in the quartz boat and then place it in a quartz tube. Push the quartz tube into a dual-temperature zone tube furnace for the reaction. Introduce Ar and O_2_ with a flow rate of 150:15 sccm and set the temperature to 1050℃. After 40 minutes, the experiment is completed. Remove the sample and cool it to room temperature. Vertically grown ZnO microrods can be observed on the Si substrate. The SEM image of a single ZnO microrod on the Si substrate is shown in Figure S1a. The ZnO microrod exhibits a hexagonal prism structure with a smooth surface, making it an ideal material for natural WGM microcavity. Figure S1b shows the emission spectrum of the ZnO microrod excited by a 325 nm femtosecond laser. The spectrum contains two emission centers: one is the exciton scattering emission at 390 nm, and the other is the ZnO defect emission at around 530 nm.

Furthermore, a single ZnO microrod is first selected from the array and placed on a silicon substrate. Next, a monolayer graphene sheet measuring 0.5 cm × 0.5 cm is transferred onto the surface of the ZnO microrod to construct a ZnO/Graphene hybrid microcavity. The specific process for transferring the monolayer graphene onto the ZnO microrod surface is as follows: Firstly, a clean silicon substrate and graphene grown on a copper substrate are prepared. The copper-based graphene is then cut into 0.5 cm × 0.5 cm pieces. After that, a uniform layer of polymethyl methacrylate (PMMA) is spun onto the cut graphene pieces as a protective layer. The copper foil-supported graphene is placed on a spin coater, and spinning for 30 s at 4000 rpm, followed by heating at 100°C for 3 to 5 min. Next, a 1:4 iron(III) nitrate aqueous solution is prepared as an etching solution, and the graphene pieces are immersed in the iron nitrate solution to undergo etching. Once the graphene completely floats on the surface of the etching solution and the copper substrate is no longer visible, a clean glass slide is used to pick up the graphene, gently inserting the substrate at an angle into deionized water. This step is repeated three to four times until the deionized water is clear. Subsequently, the graphene is transferred onto the ZnO microrod and dried at 60°C. Then, the substrate is soaked in acetone solution to remove the PMMA protective layer from the surface, followed by washing it with deionized water two to three times, and finally drying it after removal. Finally, a small ion sputtering device is used to sputter AgNPs onto the ZnO/Graphene hybrid microcavity, as well as on the silicon substrate and quartz substrate, with a sputtering current of approximately 14 mA and a chamber pressure of about 40 Pa.

By adjusting the sputtering time of AgNPs, we investigated their optical properties. Figure S2 presents the ultraviolet-visible absorption spectra of AgNPs on a quartz substrate at different sputtering times. For sputtering times of 5 s, 15 s, 30 s, 45 s, 60 s, and 75 s, the absorption peaks of the Ag nanoparticles are located at 400 nm, 440 nm, 500 nm, 536 nm, and 568 nm, respectively. Figure S2 compares the normalized absorption spectra of AgNPs at different sputtering times. By comparing the absorption spectra at each time point, it can be observed that as the sputtering time increases, the position of the absorption peaks of the AgNPs gradually redshifts. At 15 s, the absorption peak is located at 445 nm, which corresponds to the intrinsic absorption peak of AgNPs. This characteristic peak is most suitable for coupling with ZnO, thereby enhancing the optical field localization effect and ultimately achieving an enhancement in the Raman signal.


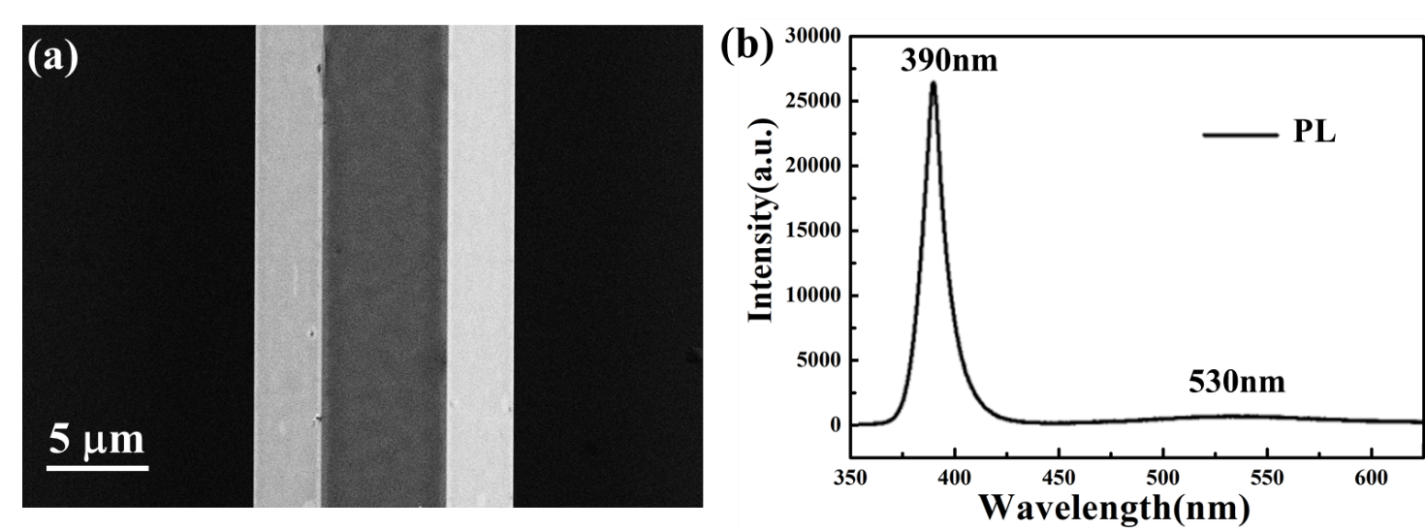


Figure S1 (a) SEM image of ZnO microrod and (b)PL spectrum of ZnO microrod


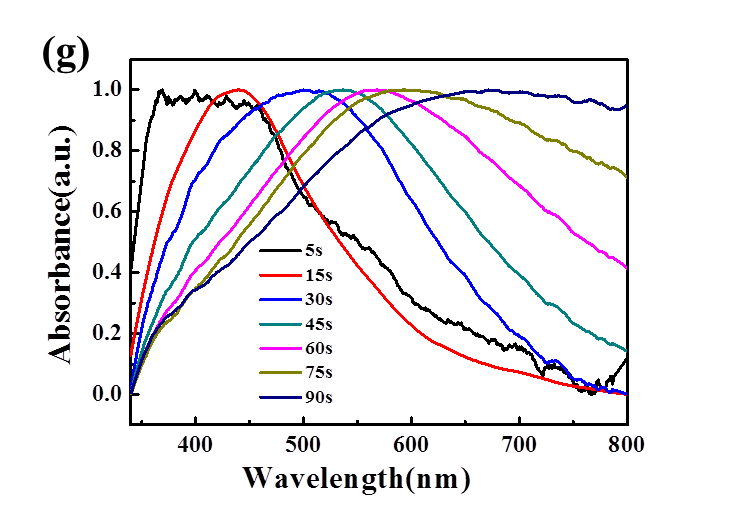


Figure S2 The absorption spectra of ZnO/AgNPs with different sputtering time (5s, 15s, 30s, 45s, 60s, 75s, 90s).


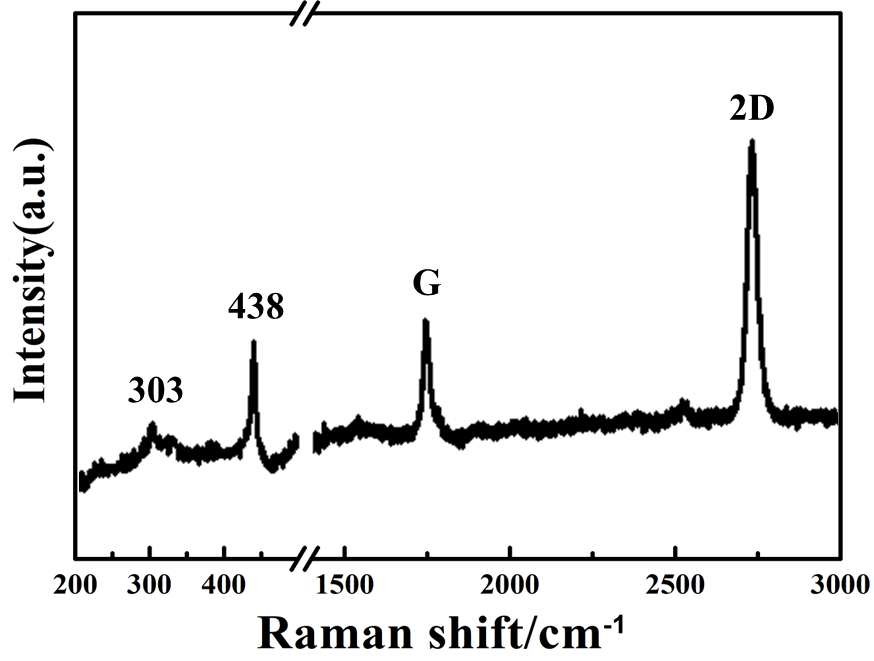


Figure S3 Raman spectra of ZnO/Graphene hybrid microcavity


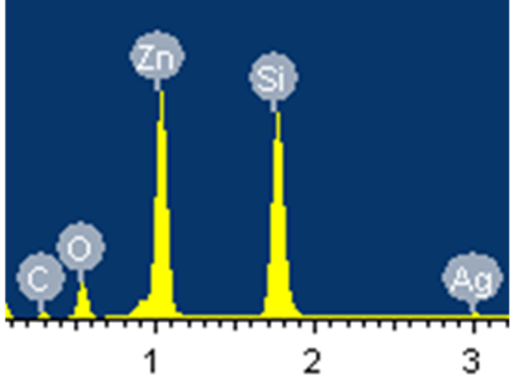


Figure S4. The EDS spectrum for the ZnO/Graphene/AgNPs hybrid microcavity


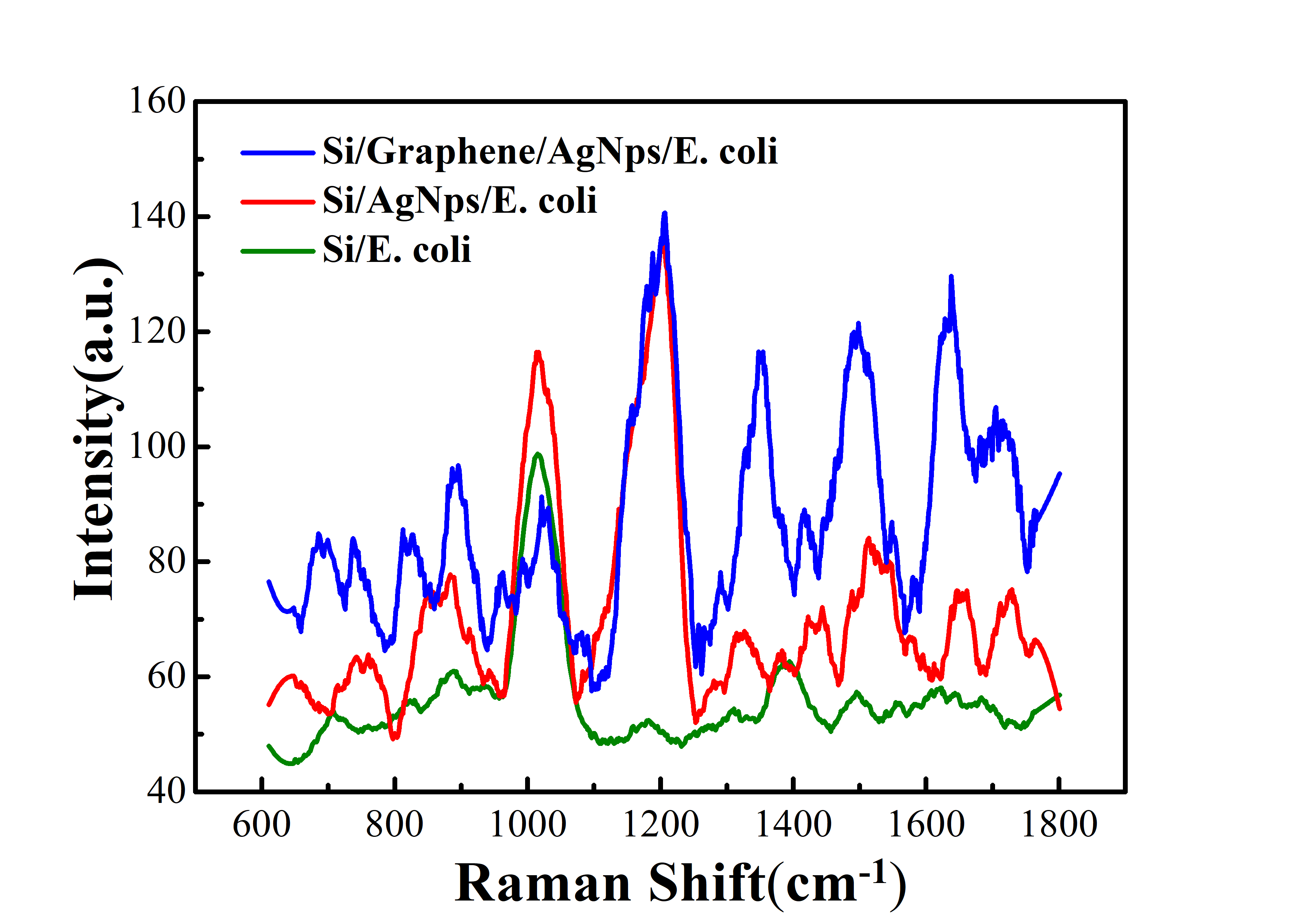


Figure S5 The amplified Raman spectra of Si, Si/AgNPs and Si/Graphene/AgNPs from Figue 2b

Figure S6 The stimulated emission spectra of ZnO, ZnO/AgNPs, and ZnO/Graphene/AgNPs

To verify the enhancement of ZnO laser performance by graphene and AgNPs and to gain a deeper understanding of the enhancement mechanism, we selected three different regions (ZnO, ZnO/Ag NPs, and ZnO/Graphene/AgNPs) for optical performance testing, with the stimulated emission spectra shown in Figure S6. In the stimulated emission spectrum of pure ZnO microrods, nine distinct peak positions can be clearly observed. Among these, the strongest resonance peak located at 393.92 nm in the emission spectrum has a full width at half maximum (FWHM) of approximately 0.112 nm, resulting in a calculated Q factor of 3517 (where Q is defined as Q = λ/Δλ, with λ being the peak wavelength and Δλ the FWHM). By comparing the stimulated emission spectra of the three structures, it was found that the intensity of the stimulated emission peak in the ZnO/ANPs hybrid microcavity increased by about 10 times compared to that of pure ZnO. Additionally, the intensity of the stimulated emission peak in the ZnO/Graphene/AgNPs hybrid microcavity was approximately twice that of the ZnO/AgNPs. Calculations showed that there is about a 20-fold enhancement in laser performance in the ZnO/Graphene/AgNPs hybrid WGM microcavity structure compared to pure ZnO. The experimental results indicate that the smooth-surfaced ZnO microrods serve as WGM microcavities, while monolayer graphene and AgNPs act as surface plasmons, enabling greater light localization at the surface of the hybrid microcavity and significantly enhancing its laser performance.


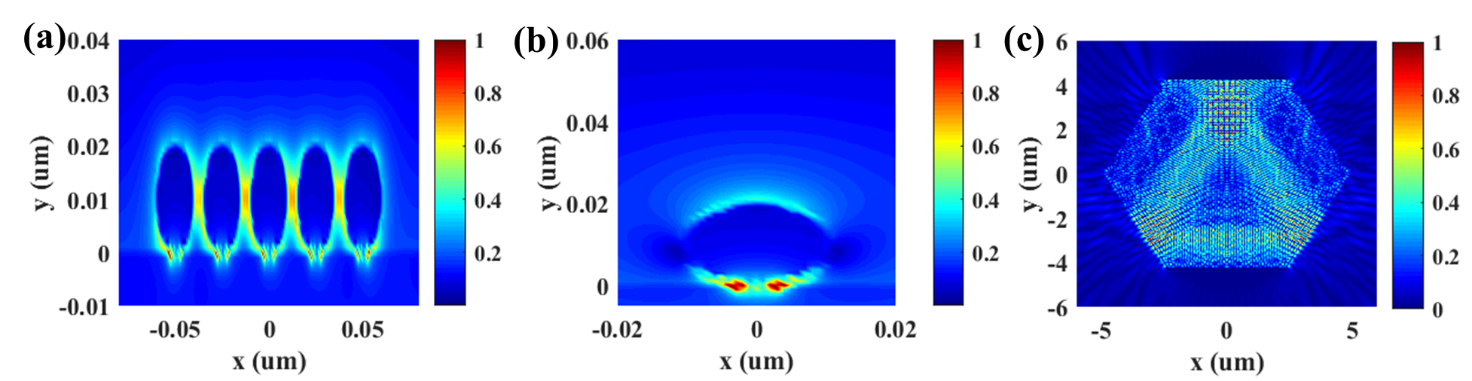


Figure S7 Electronic field intensity distributions for ZnO/Graphene/AgNPs hybrid structures.

To investigate the mechanism of Raman signal enhancement, finite-difference time-domain (FDTD) simulations were performed to validate the enhancement and localization of the local electric field by graphene and AgNPs. The optical field distribution of the hybrid microcavity was simulated, as shown in Figure S6. The parameters were set as follows: the refractive index of ZnO was 2.43, and the excitation wavelength was 532 nm. In this experiment, a single layer of graphene was first transferred to the surface of ZnO, followed by the deposition of AgNPs, which had a size of approximately 20 nm, significantly smaller than the diameter of ZnO, which is 9.805 μm. Electric field data at a wavelength of 532 nm were obtained in the planar simulation region. As shown in Figure S7, the enhancement and localization of the electric field at the interface between ZnO and AgNPs were analyzed. Due to the local surface plasmon resonance (LSPR) effect of the AgNPs, the excitation light at 532 nm was effectively confined to the ZnO interface, thereby enhancing the interaction with the target analyte molecules. The simulation results indicated that the optical field of a pure ZnO microrod primarily propagated along its surface, consistent with the total internal reflection WGM resonance mechanism of ZnO. After modification with graphene and AgNPs, more photons were localized at the surface of ZnO, significantly enhancing the optical field intensity at the ZnO surface.

Figure S8 The amplified Raman spectrum collected from a single *E. coli*
